# Iatrogenic tracheal ruptures

**DOI:** 10.1186/1749-8090-8-S2-P3

**Published:** 2013-10-23

**Authors:** B Aydemir, S Çelik, O İmamoğlu, M Çelik, T Okay

**Affiliations:** 1 Siyami Ersek Cardiothoracic Center, Istanbul, Turkey

## Background

Iatrogenic tracheal rupture is a rare, but life-threatening complication of orotracheal intubation. In this retrospective study, etiology, and diagnostic and therapeutic approaches for iatrogenic tracheal ruptures were reviewed.

## Methods

Eleven patients (6 males and 5 females) were diagnosed and treated for iatrogenic tracheal rupture in our clinic. The laceration occurred after orotracheal intubation in 7 cases, and during percutaneous tracheostomy and emergency tracheostomy in the other 4 cases, respectively. Diagnosis was made during thoracic surgery in 5 cases. The remaining cases were diagnosed in the post-operative period; the most common symptoms were mediastinal emphysema and pneumothorax.

## Results

The diagnosis was confirmed by bronchoscopy in all cases. The lacerations were longitudinal, 1-7 cm in length and were located in the distal membranous trachea. While ruptures detected intraoperatively were repaired during the surgery, the others were treated conservatively. No mortality was observed among cases treated surgically. However, two of the cases treated conservatively died (50%), and the cause of death was the underlying disease requiring intubation.

## Conclusions

We are in the opinion that primary disease is a determinant of patient outcome. Except cases identified during surgery, emergency surgical interventions should be preferred in patients, in whom ventilation cannot be achieved.

